# Recommendations to Medical Mission Trip Teams: A Retrospective Study of an Annual Medical Student-Run Mission Trip to Jarabacoa, Dominican Republic

**DOI:** 10.7759/cureus.11852

**Published:** 2020-12-02

**Authors:** Madeline MacDonald, Jessica T Phan, Liwei Chen, Taylor L Jarvill, Stephanie Yee, Paul J Wells, Rahul Mhaskar

**Affiliations:** 1 Internal Medicine, University of South Florida Morsani College of Medicine, Tampa, USA; 2 Medical Education, University of South Florida Morsani College of Medicine, Tampa, USA; 3 Pediatrics, University of South Florida Morsani College of Medicine, Tampa, USA; 4 Family Medicine, University of South Florida Morsani College of Medicine, Tampa, USA; 5 Psychiatry, University of South Florida Morsani College of Medicine, Tampa, USA

**Keywords:** global health, medical student, medical mission trip

## Abstract

Introduction

There are more than 6,000 international medical mission trips that are conducted annually by United States medical teams. Successfully planning a medical mission trip relies on careful preparation. The objective of this study is to elucidate common chief concerns, diagnoses, and prescription patterns so that medical mission trip teams can effectively prepare for future medical mission trips in Jarabacoa, Dominican Republic, or similar international sites.

Methods

A retrospective chart review of 940 patient charts was conducted from two University of South Florida Latino Medical Student Association medical mission trips to Jarabacoa, Dominican Republic (DR) that took place during October 2017 and 2018. A coding system was utilized to categorize the data. The most common chief concerns, diagnoses, and medications prescribed were revealed. Findings were stratified further by age (<18 vs ≥18 years old) and sex.

Results

Our study reveals that 68.6% (n=597/870) of the patients were female and 59.2% (n=161/870) of males were under 18. The most common chief concerns were “cold/flu” (33.2%,n=289/870), gastrointestinal problems (20.3%, n=177/870), headache (20.0%, n=174/870), and musculoskeletal problems (12.0%, n=104/870). The most common diagnoses were viral syndrome (25.4%, n=221/870), presumed parasitic infection (16.9%, n=147/870), hypertension (12.6%, n=110/870), headache (10.6%, n=92/870), and musculoskeletal disorder (8.5%, n=74/870). The most frequently prescribed medications were acetaminophen (18.3%, n=291/1,587), albendazole (15.2%, n=241/1,587), nonsteroidal anti-inflammatory drugs (NSAIDs) (10.5%, n=166/1,587), antihistamines (6.1%, n=97/1,587), and antibiotics (5.9%, n=93/1,587).

Conclusions

Our study reveals potential areas for improvement of an annual, medical student-run medical mission trip to Jarabacoa, DR. Dedicated efforts should be made to address long-term management of chronic conditions identified or treated on medical mission trips. Community partnerships should be established to facilitate this. We hope this will encourage other medical mission trip teams to analyze their data in order to be more prepared for their trips.

## Introduction

There are more than 6,000 medical mission trips that are sponsored and conducted by United States (U.S.) medical teams each year. They cost an estimated 250 million dollars according to a conservative estimate from 2008. These annual trips primarily focus on providing acute and primary care [1]. 

The University of South Florida Morsani College of Medicine (USF) Latino Medical Student Association (LMSA) has been conducting an annual medical mission trip to Jarabacoa, Dominican Republic (DR), since 2009. The DR is located on the Caribbean Island of Hispaniola and has a population of approximately 10.8 million people [2]. Most DR citizens are uninsured despite having a national healthcare system, commonly attributed to a lack of human and financial resources [3]. As of 2016, approximately 30.5% of the population lived below the international poverty line. The most common cause of death in the DR as of 2012 was ischemic heart disease, causing 18.6% of the deaths reported. This was followed by stroke (11.3%), road injury (7.5%), diabetes mellitus (4.2%), and lower respiratory infections (4.1%), respectively [4]. For medical mission trips to be sustainable and to have a positive impact on the communities they serve, it is crucial to understand the medical needs of the local community. Prior to this study, systematic documentation of the medical needs of Jarabacoa, DR, by LMSA medical mission trip members had not been previously analyzed. Medical students relied on anecdotal advice from mentors and prior medical mission trip leaders to develop the trip formulary and medical supplies list.

The purpose of this study is to elucidate the most common chief concerns, diagnoses, and prescriptions administered on LMSA medical mission trips to Jarabacoa, DR, so that we can identify areas for potential improvement and provide recommendations to future medical mission trip teams who visit the DR or similar regions.

## Materials and methods

Study sample

A retrospective chart review was conducted to collect data on chief concerns, diagnoses, prescribed medications, vital signs, and demographics from patients who obtained medical care at six medical mission trip clinic sites in October 2017 and 2018 on the LMSA medical mission trips to Jarabacoa, DR. Inclusion criteria for this study consisted of all medical records from the October 2017 and 2018 USF LMSA medical mission trips. Exclusion criteria included incomplete charts that were missing demographic data. The data collection was performed by USF medical students who led and participated in the October 2018 medical mission trip.

LMSA partners with a local Jarabacoa based community organization, that helped identify clinic sites and provided support with community outreach. The six clinic sites were visited over a three-day period by two separate LMSA medical teams. Each team was made up of 14 people that included USF attending physicians, local community physicians, nurses, medical students, and USF undergraduate student Spanish translators. Clinic sites operated from 9 a.m. to 5 p.m. Monday through Wednesday. 

Templated paper charts were utilized for the entire duration of a patient encounter and were collected upon patient check-out. All team members received a standard documentation training with detailed verbal instructions on how to systematically fill out the charts. 

Statistical analysis 

This retrospective study was approved by the USF Institutional Review Board (IRB) (Pro00037902). All medical students who participated in the study were Health Insurance Portability and Accountability Act certified prior to starting the project. Descriptive statistics were used to report the demographics, chief concerns, and diagnoses of the patients and are represented in median and range for age and frequency and percentage for other categorical variables. Analyses were conducted using statistical SAS software version 9.4 (SAS Corp., Cary, NC, USA) [5]. Chi-square test and Cochran-Mantel-Haenszel tests were performed and prevalence ratio (PR) and 95% confidence intervals (95% CIs) were calculated to investigate the association between chief concerns/diagnoses and age or sex while controlling for sex (male vs female) or age (<18 vs ≥ 18). Statistical significance was pre-specified at a two-sided p<0.05. 

The findings from this study were presented and distributed to the 2019 LMSA board members so that they could consider our recommendations. 

## Results

There were 439 and 431 participants from the mission trips in 2017 and 2018, respectively. Of the 940 total charts, 70 charts were excluded as two charts lacked age and 68 patient charts lacked sex information. 

Patient population

The patient population consisted of 31.4% (n=273/870) males and 68.7% (n= 597/870) females. Of this population 41.4% (n=360/870) were under the age of 18 and 58.6% (n=510/870) were 18 or older. Of the males 59% (n=161/273) were under the age of 18. The distribution of age was not balanced between male and female groups (p<0.0001) (Table 1). Patients ranged in age from one week to 96 years old with a median age of 28. 

**Table 1:** Patients age characteristics between male and female

	Male	Female	p-value
N	273	597	
Age			<0.0001
Youth (<18 years)	161	199	
Adult (≥18 years)	112	398	

Chief concerns

The chief concerns while controlling for age or sex can be seen in Table 2 and Table 3. Females had a higher prevalence of anemia/hematologic problems and headache than males, while males had hypertension as a chief concern more frequently than females while controlling for age (Table 3). The most common chief concerns of the overall study population were “cold” or “flu” (33.2%, n=289/870), gastrointestinal problems (20.3%, n=177/870), headache (20.0%, n=174/870), musculoskeletal problems (excluding back pain and arthritis) (12.0%, n=104/870), back pain (9.4%, n=82/870), and cough (8.2%, n=71/870). 

**Table 2:** Association between sex and chief concerns while controlling for age aPR (Female vs. Male): adjusted prevalence ratio was estimated by using Cochran-Mantel-Haenszel test.

Chief Concerns	Sex		
	Female N (%)	Male N (%)	aPR (95% CIs)
Vaginal infection	25 (4.2)	N/A	1.03 (1.02 – 1.05)
Other gynecologic problems	14 (2.3)	N/A	1.02 (1.004 – 1.03)
Anemia/Hematologic problems	13 (2.2)	0 (0)	1.02 (1.01 – 1.03)
Headache	139 (23.3)	35 (12.8)	1.10 (1.03 – 1.17)
Musculoskeletal problems			
< 18 years	8/199 (4.0)	2/161 (1.2)	1.029 (0.995 – 1.064)
≥ 18 years	65/398 (16.3)	29/112 (25.9)	0.886 (0.787 – 0.996)
Hypertension	37 (6.2)	19 (7.0)	0.96 (0.92 – 1.00)

**Table 3:** Association between age and chief concerns while controlling for sex aPR: adjusted prevalence ratio was estimated by using Cochran-Mantel-Haenszel test.

Chief Concerns	Age		aPR (95% CIs)
	<18 years N (%)	≥18 years N (%)	
“Cold” or “flu”	174 (48.3)	115 (22.5)	0.66 (0.59 – 0.74)
Gastrointestinal problems	95 (26.4)	82 (16.1)	0.87 (0.80 – 0.93)
Headache	47 (13.1)	127 (24.9)	1.14 (1.06 – 1.21)
Cough	41 (11.4)	30 (5.9)	0.94 (0.90 – 0.98)
Rash	39 (10.8)	25 (4.9)	0.93 (0.89 – 0.97)
Urological problems	3 (0.8)	25 (4.9)	1.04 (1.02 – 1.07)
Musculoskeletal problems			
Male	2/161 (1.2)	29/112 (25.9)	1.33 (1.19 – 1.49)
Female	8/199 (4.0)	65 (16.3)	1.15 (1.09 – 1.21)
Back Pain	2 (0.6)	80 (15.7%)	1.19 (1.14 – 1.24)
Hypertension	1 (0.3)	55 (10.8)	1.13 (1.09 – 1.17)
Cardiovascular problems	5 (1.4)	34 (6.7)	1.06 (1.03 – 1.09)
Diabetes	0 (0)	11 (2.2)	1.02 (1.01 – 1.03)
Dizziness/Vertigo	2 (0.6)	18 (3.5)	1.03 (1.01 – 1.05)
Eye/vision problems	6 (1.7)	24 (4.7)	1.03 (1.01 -1.06)
Medication Refill	0 (0)	20 (3.9)	1.04 (1.02 – 1.06)
Neurologic problems	0 (0)	10 (2.0)	1.02 (1.01 – 1.03)
Vaginal infection	0 (0)	25 (4.9)	1.04 (1.03 – 1.06)

Diagnoses

The most common diagnoses were viral syndrome (25.4%, n=221/870), presumed parasitic infection (16.9%, n=147/870), hypertension (12.6%, n=110/870), headache/migraines (10.6%, n=92/870), and musculoskeletal disorder (8.5%, n=74/870) (Table 4). 

**Table 4:** Diagnoses associated with age while controlling for sex aPR (Adults vs. youth): adjusted prevalence ratio was estimated by using Cochran-Mantel-Haenszel test.

Diagnoses	Age		
	<18 years N (%)	≥18 years N (%)	aPR (95% CI)
Viral Syndrome	142 (39.4)	79 (15.5)	0.70 (0.64 – 0.77)
Presumed parasitic infections	115 (31.9)	32 (6.3)	0.73 (0.68 – 0.79)
Dermatologic issues	34 (9.4)	30 (5.9)	0.96 (0.92 – 1.00)
Rash	22 (6.1)	11 (2.2)	0.96 (0.93 – 0.98)
Allergies	20 (5.6)	50 (9.8)	1.05 (1.01 – 1.09)
Headache/Migraines	18 (5.0)	74 (14.5)	1.10 (1.05 – 1.15)
Well Visit	17 (4.7)	4 (1.2)	0.96 (094 – 0.99)
Musculoskeletal Disorder (excluding arthritis and back pain)			
Male	1/161 (0.6)	21/114 (18.4)	1.22 (1.12 – 1.33)
Female	6/199 (3.0)	46/396 (11.6)	1.10 (1.05 – 1.15)
Constipation	13 (3.6)	44 (8.6)	1.05 (1.01 – 1.08)
Gastroesophageal Reflux Disease	5 (1.4)	47 (9.2)	1.08 (1.05 – 1.12)
Hypertension	4 (1.2)	106 (20.8)	1.28 (1.21 – 1.34)
Cardiovascular disorders	0 (0)	13 (2.5)	1.03 (1.01 – 1.04)
Diabetes	1 (0.3)	29 (5.7)	1.06 (1.04 – 1.09)
Back Pain	2 (0.6)	54 (10.6)	1.12 (1.08 – 1.16)
Eye/vision disorders	2 (0.6)	14 (2.7)	1.02 (1.00 -1.04)
Arthritis	2 (0.6)	38 (7.5)	1.08 (1.05 – 1.10)
Neurologic disorders	0 (0)	14 (2.7)	1.03 (1.01 – 1.04)
Kidney disorders	1 (0.3)	9 (1.8)	1.04 (1.03 – 1.06)
Hyperlipidemia/Hypocholesteremia	1 (0.3)	15 (2.9)	1.03 (1.01 – 1.05)
Costochondritis	0 (0)	6 (1.2)	1.01 (1.00 – 1.03)
Injury	1 (0.3)	10 (2.0)	1.02 (1.01 – 1.04)
Gynecologic Disorder	2 (0.6)	29 (5.7)	1.05 (1.02 – 1.07)
Respiratory Disorder	1 (0.3)	11 (2.2)	1.02 (1.01 – 1.03)

Prescriptions

The most frequently prescribed medications were acetaminophen (18.3%, n=291/1,587), albendazole (15.2%, n=241/1,587), nonsteroidal anti-inflammatory drugs (NSAIDs) (10.5%, n=(166/1,587), antihistamines (6.1%, n=97/1,587), and antibiotics (5.9%, n=93/1,587) (Table 5). 

**Table 5:** Trip Formulary *CCB=calcium channel blocker *BB=beta blocker *ARB=angiotensin 2 receptor blocker *B2 agonist=beta agonists *H1 receptor antagonist=histamine receptor 1 antagonist *H2 receptor antagonist=histamine receptor 2 antagonist *PPI=proton pump inhibitor *NSAIDs=nonsteroidal anti-inflammatory drugs

Medication Name/Type	Dosage	Drug class
Antihypertensives		
amlodipine	5 mg	*CCB
amoldipine	10 mg	CCB
bacifloxacin OPTH solution	0.6% 2mL	fluoroquinolone
carvedilol	3.125 mg	*BB
ciprofloxacin	250mg	fluoroquinolone
furosemide	20 mg	loop diuretic
hydrochlorothiazide	25 mg	thiazide diuretic
hydrochlorothiazide	50mg	thiazide diuretic
lisinopril	10mg	ACE inhibitor
lisinopril	20mg	ACE inhibitor
metoprolol succinate	50 mg	BB
metoprolol tartrate	25 mg	BB
metoprolol tartrate	50mg	BB
ofloxacin 0.3% ophthalmic 5 mL drops	0.3%/5ml	fluoroquinolone
propranolol hydrochloride	60mg	BB
telmisartan	80mg	*ARB
valsartan	80mg	ARB
verapamil	240mg	CCB
Analgesics		
acetaminophen (children’s)	80mg	analgesic
acetaminophen extra strength	500 mg	analgesic
acetaminophen solution	160mg/5ml	analgesic
aspirin	81 mg	NSAID
aspirin (chewable)	81mg	NSAID
aspirin	325mg	NSAID
diclofenac sodium	100mg	NSAID
ibuprofen	400mg	NSAID
ibuprofen	800 mg	NSAID
ibuprofen	200 mg	NSAID
ibuprofen	600 mg	NSAID
ibuprofen solution - Children	100 mg/5mL	NSAID
indomethicin	50mg	NSAID
naproxen	220 mg	NSAID
Antacids		
calcium carbonate	500 mg	antacid
Antibiotics		
azithromycin	250 mg	macrolide
amoxicillin	875mg	B-lactam
amoxicillin	500mg	B-lactam
amoxicillin (oral)	250mg/5ml	B-lactam
amoxicillin (oral)	125mg/5ml	B-lactam
ampicillin	500mg	B-lactam
bacitracin	1oz	Antibiotic
cefazolin sodium	1gm	B-lactam
ceftriaxone im 1g	1g	B-lactam
doxycycline	50gm	tetracycline
metronidazle	500mg	nitromidazole
nitrofurantoin	100mg	antibiotic
trimethoprim-sulfamethoxazole	120ml solution (40/200mg per 5 mL)	sulfonamide
trimethoprim-sulfamethoxazole	800mg/160mg	sulfonamide
Anticonvulsants		
gabapentin	400 mg	anticonvulsant
valproic acid	250mg	anticonvulsant
Antidiarrheals		
bismuth subsalicylate	262mg/8oz	antidiarrheal
loperamide	2mg	antidiarrheal
Antiemetics		
odansetron	4 mg	antiemetic
promethezine	25mg	antiemetic
Antifungals		
clotrimazole topical	1oz	antifungal
itraconazole	100mg	antifungal
itraconazole solution	10mg/ml	antifungal
miconazole nitrate powder	2% powder	antifungal
miconazole nitrate topical	250mg	antifungal
Antimalarials		
chloroquine	50mg/5ml	antimalarial
Antiparasitics		
albendazole	400mg	antiparasitic
ivermectin	6mg	antiparasitic
Antivirals		
acyclovir	200mg	antiviral
Antidiabetics		
glyburide	2.5mg	sulfonylurea
metformin	500mg	biguanide
B2 agonists		
albuterol sulfate inhalation solution	0.083% 2.5mg/3ml	*beta-2 agonist
salbutamol	100 mg/dose	beta-2 agonist
Corticosteroids		
clobetasol propionate topical 0.05%	45mg	corticosteroid
hydrocortisone anti-itch cream	1oz	corticosteroid
prednisone	5mg	corticosteroid
prednisone	10mg	corticosteroid
triamcinolone	0.10%	corticosteroid
Expectorants		
guanifensin	200mg/10mL	expectorant
Anticholesterols		
fenofibrate	160mg	fibrate
simvastatin	20mg	statin
Antihistamines		
cetirizine hydrochloride	10mg	*H1HhH1 receptor antagonist
chewable cetirizine hydrochloride	10mg	H1 receptor antagonist
diphenhydramine hydrochloride	50mg	H1 receptor antagonist
hydroxyzine	50 mg	H1 receptor antagonist
loratadine chewable	5mg	H1 receptor antagonist
meclizine hcl	25mg	H1 receptor antagonist
Antacids		
famotidine	20 mg	*H2 receptor antagonist
ranitidine	150mg	H2 receptor antagonist
Hormones		
levothyroxine	100mcg	hormone
Proton Pump Inhibitors		
esomeprozole magnesium	22.3 mg	*PPI
lansoprazole	30mg	PPI
omeprazole	20mg	PPI
Laxatives		
docusate calcium	100mg	stool softener
docusate sodium	100mg	stool softener
sennosides	8.6mg	stimulant laxative

## Discussion

Our study reveals potential areas for improvement of an annual, medical student-run medical mission trip to Jarabacoa, DR. Women and children made up the majority of the study population. A possible explanation could be that clinics were open during business hours, which could have been a potential barrier for working men to access care. Our findings are in agreement with a 2014 study, therefore it is possible that other medical mission trips may encounter this same disparity [6]. We therefore recommend that future medical mission trip teams consider this and consider implementing weekend or evening clinics. 

There is debate about appropriate standard of care for medical mission trips. A systematic review performed in 2017 found a lack of consensus and clarity between recommendations for medical mission trips with regards to quality of care [7]. Some researchers recommend providing equivalent standard of care as at-home institutions while others encourage following a local standard of care. Others recommended adjusting standard of care from the home institution based on the resources available at the local site [7]. With this in mind, we discuss screening and treatment for hypertension and diabetes in the context of the U.S. Preventative Task Force (USPTF) guidelines [8]. These are evidence-based guidelines practiced at USF that clinicians at the LMSA clinics aimed to follow as closely as possible given the local resources. While the subject of standard of care for medical mission trips remains an ethical debate, clear recommendations on how best to ensure the delivery of quality care in a medical mission trip setting remains open to interpretation. We recommend that each medical mission trip team follows evidence-based guidelines when providing medical care internationally. 

Hypertension

Chronic diseases including hypertension were frequently evaluated in the LMSA clinics, which is consistent with the high prevalence of hypertension in the DR. Nearly 35% of Dominicans have hypertension [6]. Of note, approximately 12% of our study participants had hypertension. While some patients were found to have elevated blood pressure readings during the clinic visit, it was not possible to diagnose new cases of hypertension based on a single blood pressure reading per USPTF guidelines. Anti-hypertensive medications were not routinely prescribed in LMSA clinics unless the patient needed a refill for a preexisting prescription. Clinicians voiced their concerns that patients would not have access to antihypertensive medication refills once the limited supply ran out, therefore putting these patients at risk for rebound hypertension. To respond to this care gap we recommend that future medical mission trip teams have dedicated board members whose role is to create partnerships with DR providers and government agencies in order to provide patients with follow-up care and medication refills. 

Diabetes

The DR has an estimated diabetes prevalence of approximately 11% [9]. We found that approximately 3% of our study participants had diabetes. When stratified by age, 5.7% of adults had a diagnosis of diabetes, and 0.02% of patients under 18 had diabetes. Based on the lower than expected prevalence of diabetes in our clinic population, we suspect that diabetes is underdiagnosed and recommend that future medical mission trip teams routinely screen all individuals who meet screening criteria based on the USPTF guidelines [10]. 

It is important to recognize how a new diagnosis of a chronic disease such as diabetes or hypertension might affect patients, with special consideration to financial, logistical, or geographical barriers to receiving healthcare. Literature suggests that patients may be aware that they need to take medication(s) for conditions that they have been diagnosed with, but reliable access to affordable medications may be the limiting factor [11]. We strongly recommend that these patients are referred to physicians in the community so that they can continue to receive care and prescription refills. 

The most commonly distributed medications in the LMSA clinics included acetaminophen, albendazole, NSAIDs, antihistamines, and antibiotics. These prescribed medications were consistent with the diagnoses made. With this said, the selection of medications by clinicians at LMSA clinics were limited to what was available in the formulary (Table 5). Medications in the formulary were obtained through donations from national organizations, donations from U.S. pharmacies, as well as purchases from U.S. and DR pharmacies. This information is critical to advise future trips on which medications to include in the formulary. The 2018 formulary was provided to the 2019 LMSA board members so that they could adjust the formulary as necessary and help reduce unnecessary waste and cost. For example, based on the 2018 data, there was an excess of chloroquine, metformin, and anti-hypertensives but a shortage of amoxicillin and NSAIDs. We recommend that medical mission trip teams similarly maintain a database to better prepare for their future medical mission trips. 

Limitations

There are limitations to this study which include the sample size, selection bias, and the retrospective nature of the study. Additionally, we were unable to distinguish patients who returned to the clinic from 2017 and 2018. Age groups were not further stratified due to limited sample size. Furthermore, certain diseases were grouped into disease categories due to limited sample size. We did not conduct a risk factor assessment as our data was limited to what was documented in the medical records. Future studies could benefit from risk factor assessment to help prevent and manage chronic disease in medical mission trip clinics. When reviewing charts, we are limited to what information was documented in the charts. Errors may have occurred during Spanish to English translation, leading to potential inaccuracy. Additionally, gender was not specifically elucidated so further studies could benefit from identifying transgender or non-binary patients. 

## Conclusions

This study reveals areas for potential improvement on a medical student run medical mission trip to Jarabacoa, DR. As there is limited literature available on medial mission trip planning, our study reveals the utility of analyzing medical record data to more effectively prepare for medical mission trips to Jarabacoa and similar international sites. Evidence based guidelines should be used to screen and treat patients abroad, whenever possible. Local, sustainable solutions are needed for appropriate management of chronic conditions identified or treated on medical mission trips. Future studies could propose interventions to help mitigate and better manage chronic diseases treated in medical mission trip clinics. 

## References

[1] Maki J, Qualls M, White B, Kleefield S, Crone R (2008). Health impact assessment and short-term medical missions: a methods study to evaluate quality of care. BMC Health Serv Res.

[2] (2019). Dominican Republic | Data. https://data.worldbank.org/country/dominican-republic.

[3] Canario JA, Lizardo J, Espinal R, Colomé M (2016). Gaps in health research in the Dominican Republic. Rev Panam Salud Publica.

[4] (2019). Global Health - Dominican Republic. https://www.cdc.gov/globalhealth/countries/dr/default.htm.

[5] (2014). SAS [Computer Program] Version 9.4. Version 9.4. Cary, NC: SAS Institute Inc.

[6] Ferrara BJ, Townsley E, MacKay CR, Lin HC, Loh LC (2014). Short-term global health education programs abroad: disease patterns observed in Haitian migrant worker communities around La Romana, Dominican Republic. Am J Trop Med Hyg.

[7] Roche SD, Ketheeswaran P, Wirtz VJ (2017). International short-term medical missions: a systematic review of recommended practices. Int J Public Health.

[8] (2020). Final Recommendation Statement: High Blood Pressure in Adults: Screening. https://www.uspreventiveservicestaskforce.org/Page/Document/RecommendationStatementFinal/high-blood-pressure-in-adults-screening.

[9] Dethlefs HJ, Walker EA, Schechter CB (2019). Evaluation of a program to improve intermediate diabetes outcomes in rural communities in the Dominican Republic. Diabetes Res Clin Pract.

[10] (2020). Final Update Summary: Abnormal Blood Glucose and Type 2 Diabetes Mellitus: Screening - US Preventive Services Task Force. https://www.uspreventiveservicestaskforce.org/Page/Document/UpdateSummaryFinal/screening-for-abnormal-blood-glucose-and-type-2-diabetes.

[11] Castro B, Ing L, Park Y, Abrams J, Ryan M (2018). Addressing noncommunicable disease in Dominican Republic: barriers to hypertension and diabetes care. Ann Glob Health.

